# Vereinbarkeit von Familie und Beruf für Ärztinnen in Deutschland am Beispiel der Urologie

**DOI:** 10.1007/s00120-024-02439-8

**Published:** 2024-09-16

**Authors:** Laura Wiemer, Sophie Knipper, Annika Herlemann, Maria-Noemi Welte, Carolin Siech, Eva-Maria Greiser, Karina Müller, Laura Bellut, Sandra Schönburg, Margarete Walach, Raisa Pompe, Sarah Weinberger

**Affiliations:** 1 Klinik für Urologie, Charité – Universitätsmedizin Berlin, Berlin, Deutschland; 2 Klinik für Urologie, Vivantes Klinikum am Urban, Berlin, Deutschland; 3 Klinik für Urologie, Campus Großhadern, LMU München, München, Deutschland; 4 Universitätsklinikum, Klinik für Urologie, Goethe-Universität Frankfurt, Frankfurt am Main, Deutschland; 5 Klinik für Urologie, Mathias-Spital, Hospital Rheine, Rheine, Deutschland; 6 Klinik für Urologie, Klinikum Bremen Mitte, Bremen, Deutschland; 7 Klinik für Urologie und Kinderurologie, Universitätsklinikum Erlangen, Erlangen, Deutschland; 8 BG Klinikum Bergmannstrost, Halle (Saale), Deutschland; 9 Klinik für Urologie und Urochirurgie, Universitätsmedizin Mannheim (UMM), Universität Heidelberg, Mannheim, Deutschland; 10 Klinik für Urologie, Klinikum Herford, Herford, Deutschland

**Keywords:** Geschlechtergerechtigkeit, Gesundheitswesen, Berufliche Chancengleichheit, Karrierehindernisse, Gender equality, Healthcare system, Equal career opportunities, Career barriers

## Abstract

**Hintergrund:**

Der Fachkräftemangel in der Medizin stellt eine der drängendsten Herausforderungen im Gesundheitswesen dar. Die steigende Anzahl von Frauen in der Medizin und im Speziellen in dem Fachbereich der Urologie wirft Fragen zur Vereinbarkeit von Familie und Beruf auf, insbesondere in Bezug auf die Arbeitsumgebung und Arbeitszeitmodelle.

**Ziel der Arbeit:**

Ziel der Arbeit ist es, die Auswirkungen von Mutterschaft auf das Berufsleben von Ärztinnen und Wissenschaftlerinnen im Fachbereich Urologie in Deutschland zu erfassen. Dabei sollen spezifische Herausforderungen in diesem chirurgischen Fachgebiet sowie die Vereinbarkeit von Familie und Beruf beleuchtet werden.

**Methode:**

Die Arbeitsgemeinschaft „Ärztinnen und Wissenschaftlerinnen in der Urologie“ der Deutschen Gesellschaft für Urologie (DGU) e. V. befragte ihre 1343 weiblichen Mitglieder zu demografischen Daten, beruflichem Status und Aspekten der Vereinbarkeit von Familie und Beruf.

**Ergebnisse:**

Unter 487 Urologinnen in Deutschland hatten 53,4 % Kinder. Mütter waren tendenziell älter, seltener in Weiterbildung, seltener im stationären Bereich und seltener operativ tätig. Besonders auffällig war der geringe Anteil von Vollzeit-arbeitenden Müttern (36,2 %) im Vergleich zu den Urologinnen ohne Kinder (92,4 %). Von den Urologinnen mit Kindern gaben 32,3 % an, ihre Arbeitsstätte wegen ihrer Kinder gewechselt zu haben, während 10,7 % angaben, dass sich nach einer Schwangerschaft zumindest ihr Aufgabenbereich geändert hat. Darüber hinaus reduzierten 76,9 % der Mütter ihre Wochenarbeitsstunden aufgrund familiärer Verpflichtungen. In der multivariablen Analyse zeigte sich ein Einfluss von Mutterschaft auf beruflichen Status und Teilzeitarbeit.

**Schlussfolgerung:**

Die Tatsache, dass die Gründung einer Familie für Frauen in der Urologie in Deutschland mit einer Arbeitszeitreduktion und Beendigung der Klinikkarriere einhergeht, legt nahe, dass es in Deutschland die Notwendigkeit einer Optimierung der Vereinbarkeit von Familie und Beruf gibt. Durch die zunehmende Feminisierung des Medizinberufs wird der bestehende Fachkräftemangel durch das Ausscheiden von Müttern aus dem Berufsleben verstärkt. Um den Bedürfnissen von berufstätigen Eltern, insbesondere Müttern, gerecht zu werden, sind daher dringend Anpassungen im Arbeitsumfeld notwendig. Die Förderung flexibler Arbeitszeitmodelle und die Schaffung unterstützender Rahmenbedingungen sind entscheidend, um den Verlust von Fachkräften zu vermeiden und die Arbeitszufriedenheit in diesem Bereich zu erhalten.

**Graphic abstract:**

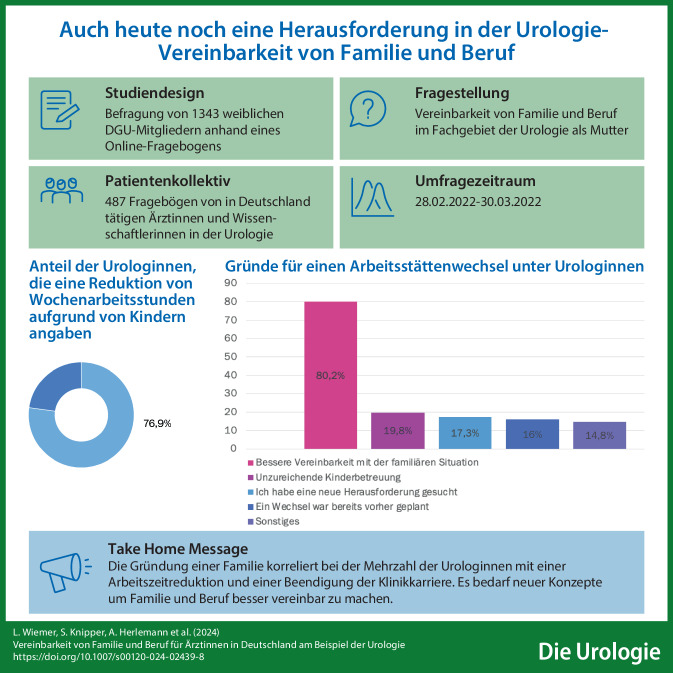

## Abstract (en)

**Background:**

The shortage of skilled labor in medicine is one of the most pressing challenges in healthcare. The increasing number of women in medicine, particularly in the field of urology, raises questions about the compatibility of family and career, especially concerning the work environment and working time models.

**Objective:**

The aim of this study is to capture the impact of motherhood on the professional lives of female physicians and scientists in the field of urology in Germany. Specific challenges in this surgical specialty and the compatibility of family and career will be highlighted.

**Methods:**

The working group “Female Physicians and Scientists in Urology” of the German Society of Urology (DGU) surveyed its 1343 female members regarding demographic data, professional status, and aspects of work–life balance.

**Results:**

Among 487 female urologists in Germany, 53.4% had children. Mothers tended to be older, less frequently in training, less often in inpatient settings, and less frequently engaged in surgical activities. Notably, the proportion of full-time working mothers (36.2%) was significantly lower compared to female urologists without children (92.4%). Among female urologists with children, 32.3% reported having changed their workplace because of their children, while 10.7% indicated that their responsibilities had changed at least once after pregnancy. Additionally, 76.9% of mothers had reduced their weekly working hours due to family commitments. Multivariate analysis showed an influence of motherhood on professional status and parttime work.

**Conclusion:**

The fact that starting a family for women in urology in Germany is associated with a reduction in working hours and the end of clinical careers suggests a need for optimizing the compatibility of family and career in Germany. The increasing feminization of the medical profession exacerbates the existing shortage of skilled workers due to the exit of mothers from professional life. To meet the needs of working parents, particularly mothers, urgent adjustments in the work environment are necessary. Promoting flexible working time models and creating supportive conditions are crucial to preventing the loss of skilled professionals and maintaining job satisfaction in this field.

## Hintergrund und Fragestellung

Der Fachkräftemangel in der Medizin stellt eine der drängendsten Herausforderungen im Gesundheitswesen dar. Besonders auffällig ist dabei die wachsende Bedeutung von Frauen in der Medizin. Nicht nur die Mehrheit der Studierenden der Medizin sind weiblich [1], sondern auch der Anteil von Frauen im ärztlichen Beruf nimmt stetig zu. Derzeit sind rund 40 % der berufstätigen Ärzte weiblich. Dies zeigt sich auch in dem traditionell männerdominierten Fachbereich der Urologie.

### Der Anteil der berufstätigen Ärztinnen in der Urologie nimmt stetig zu

Der Anteil der berufstätigen Ärztinnen in der Urologie nimmt stetig zu und lag im Jahr 2022 bei 21,7 %. Im Jahr 2008 lag der Frauenanteil noch bei 11,3 % [2]. Trotz dieser positiven Entwicklung zeigt sich weiterhin eine Schere zwischen Männern und Frauen: Auf Leitungspositionen sind Frauen unterrepräsentiert. So sind nur 13 % der Klinikdirektoren weiblich [3]. Die Vereinbarkeit von Familie und Beruf scheint in diesem Zusammenhang eine große Rolle zu spielen. In einer Studie zu Auswirkungen der Elternzeit auf Karriereziele an einer medizinischen Hochschule wurde belegt, dass 51 % der Beschäftigten nach Rückkehr aus der Elternzeit eine signifikante Änderung ihrer Arbeitsaufgaben erlebten, 17 % der Führungskräfte ihren Status verloren und 58 % einen Arbeitgeberwechsel erwogen [4]. Nach wie vor nehmen Frauen deutlich häufiger Elternzeit in Anspruch und arbeiten auch oft danach in Teilzeit [5].

### Vereinbarkeit von Familie und Beruf als Balanceakt

In der Medizin kommen zudem besondere Herausforderungen auf Eltern zu. Dazu gehört neben langen Arbeitszeiten die Arbeit nachts, am Wochenende und an Feiertagen. Trotz zunehmender Digitalisierung im Arztberuf kann die Arbeit selten von zu Hause erledigt werden. Es sind keine flexiblen Arbeitszeiten gegeben und oftmals kollidieren Arbeitszeiten mit Öffnungszeiten der Kinderbetreuungseinrichtungen. Soziale Erwartungen des Umfelds bezüglich der Rolle von Frauen in der Familie können zusätzlichen Druck auf Ärztinnen ausüben. Fuß et al. stellten in einer Studie mit 296 Ärztinnen und Ärzten im Krankenhaus fest, dass es bei ihnen im Vergleich zur Allgemeinbevölkerung einer signifikant erhöhten Rate an Arbeits-Familien-Konflikten kommt, welche zu Stress, Unzufriedenheit und Kündigungsabsichten führen [6].

Die vorliegende Arbeit der Arbeitsgemeinschaft (AG) „Ärztinnen und Wissenschaftlerinnen in der Urologie“ der Deutschen Gesellschaft für Urologie e. V. (DGU) untersucht die Auswirkungen von Mutterschaft auf das Berufsleben von Ärztinnen und Wissenschaftlerinnen, die in Deutschland im Fachbereich der Urologie tätig sind. Beleuchtet werden sollen dabei spezifische Herausforderungen in dem chirurgischen Fachgebiet sowie Aspekte der Vereinbarkeit von Familie und Beruf.

## Methodik

### Weibliche DGU-Mitglieder wurden befragt

Im Rahmen dieser Arbeit wurden Antworten einer Befragung der weiblichen DGU-Mitglieder ausgewertet. Der Link zum Online-Fragebogen wurde am 28.02.2022 über die E‑Mail-Verteilerliste der DGU an insgesamt 1343 weibliche DGU-Mitglieder verschickt und bis zum 30.03.2022 von insgesamt 521 Ärztinnen beantwortet. Aufgrund deutlich anderer Arbeitssituationen für Mütter, wurden die Antworten von 34 Ärztinnen aus Österreich und der Schweiz ausgeschlossen, so dass insgesamt die Antworten von 487 Ärztinnen ausgewertet wurden. Von insgesamt 43 Fragen zu u. a. demografischen Daten, Berufstätigkeit und Karriere, bezog sich fast die Hälfte der Fragen auf Aspekte zur Vereinbarkeit von Familie und Beruf. So wurden beispielsweise explizit die familiäre Situation, Alter der Kinder, Arbeitssituation des Partners und Elternzeiten erfragt.

Zu den deskriptiven Statistiken gehörten Häufigkeiten und Proportionen für kategoriale Variablen. Für kontinuierlich kodierte Variablen wurden Mittelwerte, Mediane und Interquartilsabstände angegeben. Die statistische Signifikanz von Unterschieden in den Medianen und Proportionen wurde mit den Kruskal-Wallis- und χ^2^-Tests bewertet. Univariable und multivariable logistische Regressionsmodelle untersuchten die Beziehung zwischen familiärer Situation (Kinder nein vs. ja) und verschiedenen Variablen, wie Alter (kontinuierlich kodiert), Partnerschaftsstatus (Partner/Partnerin ja vs. nein), beruflicher Status (Weiterbildung vs. Fachärztin vs. Oberärztin/leitende Ärztin/Chefärztin vs. Praxis), Arbeitszeitmodell (Vollzeit vs. Teilzeit), Promotion (ja vs. nein), wissenschaftliche Tätigkeit (ja vs. nein) und operative Tätigkeit (ja vs. nein). Die Prädiktoren wurden aus potenziellen Faktoren ausgewählt, die bereits zuvor als zusammenhängend beschrieben worden waren. Sie wurden in die multivariablen Modelle aufgenommen, wenn sie in der univariablen Analyse signifikant mit dem Ergebnis assoziiert waren. Für alle statistischen Analysen wurde die R‑Softwareumgebung für statistische Berechnungen und Grafiken (Version 3.4.3) verwendet. Alle Tests waren zweiseitig und das Signifikanzniveau wurde auf *p* < 0,05 festgelegt.

## Ergebnisse

In der Befragung gaben 260 von insgesamt 487 Urologinnen (53,4 %) an, Kinder zu haben.

### Charakteristika der Urologinnen mit und ohne Kinder

Die Urologinnen mit Kindern waren signifikant älter (41 vs. 33 Jahren) und befanden sich signifikant häufiger in einer Partnerschaft (91,9 vs. 74,4 %). Zudem befanden sich Urologinnen mit Kindern signifikant länger in Weiterbildungszeit (6,0 vs. 5,5 Jahre; Tab. 1).

**Tab. 1:** Charakteristika der Gesamtkohorte

Variable		Overall	Kinder = Ja	Kinder = Nein	*p* t/chi
			260 (53,4)	224 (46,0)	
Alter (Jahre)	Median (IQR)	37 (33–44)	41 (36–48)	33 (30–38)	< 0,001
Alter (Jahre, kategorial)	≤ 30 Jahre	68 (14)	3 (1,2)	65 (29)	< 0,001
	31–40 Jahre	238 (48,9)	126 (48,5)	112 (50)	
	41–50 Jahre	100 (20,5)	80 (30,8)	19 (8,5)	
	51–60 Jahre	66 (13,6)	42 (16,2)	24 (10,7)	
	> 60 Jahre	13 (2,7)	8 (3,1)	3 (1,3)	
	Keine Angabe	2 (0,4)	1 (0,4)	1 (0,4)	
Partnerschaft	Ja	406 (83,4)	239 (91,9)	166 (74,1)	< 0,001
	Nein	80 (16,4)	21 (8,1)	58 (25,9)	
Jahre bis zum Facharzt	Median (IQR)	6 (5–7)	6 (5–7,2)	5,5 (5–6)	0,0003

*IQR* Interquartilsabstand

Urologinnen mit Kindern befanden sich signifikant seltener noch in Weiterbildung (18,1 vs. 51,8 %) und waren seltener im stationären Sektor tätig (60,0 vs. 84,4 %). Dementsprechend waren Urologinnen mit Kindern auch seltener operativ tätig (31,5 vs. 40,6 %). Zudem gaben Urologinnen mit Kindern signifikant seltener an in Vollzeit tätig zu sein (36,2 vs. 92,4 %). Hingegen nur 7,1 % der Urologinnen ohne Kinder gaben an in Teilzeit tätig zu sein (Tab. 2).

**Tab. 2:** Angaben von Urologinnen mit und ohne Kinder, die an der Umfrage der Arbeitsgemeinschaft Urologinnen der Deutschen Gesellschaft für Urologie e. V. (DGU) teilgenommen haben

Variable	Gesamtkohorte (*n* [%])	Kinder = Ja (*n* [%])	Kinder = Nein (*n* [%])	*p*-Wert
*Wie ist Ihr beruflicher Status?*				
Weiterbildung	163 (33,5)	47 (18,1)	116 (51,8)	< 0,001
Fachärztin	137 (28,1)	92 (35,4)	45 (20,1)	< 0,001
Oberärztin/leitende Ärztin	95 (19,5)	52 (20)	43 (19,2)	< 0,001
Chefärztin	5 (1)	3 (1,2)	1 (0,4)	< 0,001
Praxis	81 (16,6)	63 (24,2)	17 (7,6)	< 0,001
Keine Angabe	6 (1,2)	3 (1,2)	2 (0,9)	< 0,001
*In welchem Sektor arbeiten Sie zurzeit? (Mehrfachantworten möglich)*				
Ambulant	169 (34,7)	119 (45,8)	48 (21,4)	< 0,001
Stationär	347 (71,3)	156 (60)	189 (84,4)	< 0,001
Industrie	7 (1,4)	7 (2,7)	0 (0)	0,03
Behörde	8 (1,6)	5 (1,9)	3 (1,3)	0,8
Andere	1 (0,2)	1 (0,4)	0 (0)	0,5
*In welchem Arbeitszeitmodell arbeiten Sie?*				
Vollzeit	303 (62,2)	94 (36,2)	207 (92,4)	< 0,001
Teilzeit	167 (34,3)	151 (58,1)	16 (7,1)	< 0,001
Derzeit nicht berufstätig	12 (2,5)	11 (4,2)	1 (0,4)	< 0,001
Keine Angabe	5 (1)	4 (1,5)	0 (0)	< 0,001
*Sind Sie operativ tätig?*				
Ja	174 (35,7)	82 (31,5)	91 (40,6)	< 0,001
Nein	110 (22,6)	83 (31,9)	26 (11,6)	< 0,001
Keine Angabe	203 (41,7)	95 (36,5)	107 (47,8)	< 0,001
*Wenn ja, operieren Sie auch komplexe Eingriffe als erste Operateurin?*				
Ja	75 (15,4)	34 (13,1)	41 (18,3)	0,5
Nein	322 (66,1)	159 (61,2)	162 (72,3)	0,5
*Sind Sie promoviert?*				
Ja	334 (68,6)	190 (73,1)	142 (63,4)	< 0,001
Nein	91 (18,7)	55 (21,2)	36 (16,1)	< 0,001
Nein, ich arbeite zurzeit noch an meiner Promotion	61 (12,5)	15 (5,8)	46 (20,5)	< 0,001
Keine Angabe	1 (0,2)	0 (0)	0 (0)	< 0,001
*Sind Sie habilitiert?*				
Ja	25 (5,1)	14 (5,4)	10 (4,5)	0,009
Nein, ich arbeite zurzeit noch an meiner Habilitation	50 (10,3)	17 (6,5)	33 (14,7)	0,009
Nein, ich strebe keine Habilitation an	401 (82,3)	226 (86,9)	174 (77,7)	0,009
Keine Angabe	11 (2,3)	3 (1,2)	7 (3,1)	0,009
*Gehen Sie aktuell einer wissenschaftlichen Aktivität nach?*				
Ja	117 (24)	47 (18,1)	69 (30,8)	0,001
Nein, ich möchte zukünftig wissenschaftlich tätig sein	48 (9,9)	21 (8,1)	27 (12,1)	0,001
Nein, ich strebe keine wissenschaftliche Tätigkeit an	318 (65,3)	190 (73,1)	127 (56,7)	0,001
Keine Angabe	4 (0,8)	2 (0,8)	1 (0,4)	0,001

Bezüglich wissenschaftlicher Aktivität gaben signifikant mehr Urologinnen mit Kindern an, promoviert zu sein (73,1 vs. 63,4 %). Jedoch gaben deutlich weniger Urologinnen mit Kindern an, aktuell wissenschaftlich aktiv zu sein (18,1 vs. 30,8 %; Tab. 2).

### Charakteristika der Urologinnen mit Kindern

Von den 260 Urologinnen mit Kindern gaben 84 (32,3 %) an, dass sie ihre Arbeitsstätte aufgrund der Kinder gewechselt hätten, 52 (10,7 %) weitere gaben an, dass sich ihr Aufgabenbereich geändert hätte. Eine Reduktion der Wochenarbeitsstunden aufgrund von Kindern gaben 200 Frauen (76,9 %) an (Tab. 3).

**Tab. 3:** Angaben von Urologinnen mit Kindern, die an der Umfrage der Arbeitsgemeinschaft Urologinnen der Deutschen Gesellschaft für Urologie e. V. (DGU) teilgenommen haben

*Haben Sie wegen des Kindes/der Kinder, Ihre Arbeitsstätte gewechselt? (n [%])*	
Ja, ich bin nicht mehr berufstätig	3 (1,2)
Ja, ich habe im Patientenfernen Bereich (z. B. Industrie) gearbeitet	8 (3,1)
Ja, ich habe in den ambulanten Bereich gewechselt	64 (24,6)
Ja, ich habe in den stationären Bereich gewechselt	9 (3,5)
Nein, ich habe bei derselben Arbeitsstätte gearbeitet, aber meine Aufgaben wurden andere	51 (19,6)
Nein, ich habe genauso gearbeitet wie vorher	121 (46,5)
Keine Angabe	4 (1,5)
*Haben Sie wegen des Kindes/der Kinder Wochenarbeitsstunden reduziert?*	
Ja	200 (76,9)
Nein	57 (21,9)
Keine Angabe	3 (1,2)

### Uni- und multivariable Analysen

In univariablen logistischen Regressionsmodellen waren das Alter (Odds Ratio [OR]: 1,1; 95 %-Konfidenzintervall (95 %-KI) 1,07–1,13), eine nicht vorhandene Partnerschaft (OR: 0,3; 95 %-KI: 0,1–0,4), der berufliche Status (Fachärztin OR: 5,1; 95 %-KI: 3,1–8,3, Oberärztin/leitende Ärztin/Chefärztin (OR: 3,1; 95 %-KI: 1,8–5,2), Praxis OR 9,1; 95 %-KI: 5,0–17,6), Arbeitszeitmodell (OR: 20,8; 95 %-KI: 12,1–38,0), Promotion (OR: 0,6; 95 %-KI:0,4–0,9), wissenschaftliche Tätigkeit (OR: 2,0; 95 %-KI:1,3–3,1) und operative Tätigkeit (OR: 3,5; 95 %-KI: 2,1–6,1) Prädiktoren für das Vorhandensein von Kindern. Ob eine Urologin habilitiert war, war kein Prädiktor bzgl. des Vorhandensein von Kindern (OR: 0,8; 95 %-KI:0,4–1,9).

In multivariablen logistischen Regressionsmodellen waren der Partnerschaftsstatus (keine Partnerschaft OR: 0,2 [95 %-KI: 0,1–0,5]), der berufliche Status (Praxis OR: 9,2 [95 %-KI: 2,7–33,8]) sowie das Arbeitszeitmodell (Teilzeit OR: 17,4 [95 %-KI: 8,0–41,6]) unabhängige Prädiktoren für eine Mutterschaft von Ärztinnen und Wissenschaftlerinnen in der Urologie (Tab. 4). Umgekehrt waren Kinder in der multivariablen Analyse nicht mit dem beruflichen Status oder der operativen Tätigkeit assoziiert, ebenso wenig wie das Erreichen einer Promotion oder Habilitation.

**Tab. 4:** Logistische Regressionsmodelle zur Vorhersage einer Mutterschaft

	Univariables logistisches Regressionsmodell				Multivariables logistisches Regressionsmodell			
Variable	OR	KI 2,5 %	KI 97,5 %	*p*-Wert	OR	KI 2,5 %	KI 97,5 %	*p*-Wert
*Wie alt sind Sie? (kontinuierlich kodiert)*	1,1	1,07	1,13	< 0,001	1,02	0,98	1,07	0,3
*Haben Sie einen Partner/eine Partnerin?*								
Ja	Ref	–	–	–	Ref	–	–	–
Nein	0,3	0,1	0,4	< 0,001	0,2	0,1	0,5	< 0,01
*Wie ist Ihr beruflicher Status?*								
In der Weiterbildung	Ref	–	–	–	Ref	–	–	–
Fachärztin	5,1	3,1	8,3	< 0,001	1,5	0,6	4,1	0,4
Oberärztin/leitende Ärztin/Chefärztin	3,1	1,8	5,2	< 0,001	2,4	0,8	7,1	0,1
In der Praxis tätig	9,1	5,0	17,6	< 0,001	9,2	2,7	33,8	< 0,001
*In welchem Arbeitszeitmodell arbeiten Sie?*								
Vollzeit	Ref	–	–	–	Ref	–	–	–
Teilzeit	20,8	12,1	38,0	< 0,001	17,4	8,0	41,6	< 0,001
*Sind Sie promoviert?*								
Ja	Ref	–	–	–	Ref	–	–	–
Nein	0,6	0,4	0,9	0,02	0,5	0,2	1,1	0,09
*Sind Sie wissenschaftlich tätig?*								
Ja	Ref	–	–	–	Ref	–	–	–
Nein	2,0	1,3	3,1	0,001	0,8	0,3	1,8	0,6
*Sie sind habilitiert?*								
Ja	Ref	–	–	–	–	–	–	–
Nein	0,8	0,4	1,9	0,7	–	–	–	–
*Sie sind operativ tätig?*								
Ja	Ref	–	–	–	Ref	–	–	–
Nein	3,5	2,1	6,1	< 0,001	1,5	0,7	3,2	0,3

*OR* Odds Ratio, *KI* Konfidenzintervall, *Ref.* Referenz

## Diskussion

### Späte Mutterschaft

Unsere Daten zeigen interessante Aspekte der Vereinbarkeit von Familie und Beruf unter Ärztinnen und Wissenschaftlerinnen in Deutschland. Sie bestätigen den weltweiten Trend, dass Frauen ihre Kinder immer später im Leben bekommen [7]. In Deutschland stieg beispielsweise das mittlere Alter der Frauen bei der Geburt des ersten Kindes von 24 Jahren im Jahr 1970 auf 28,5 Jahre im Jahr 2008 [8]. In unserer Befragung waren von den 260 Urologinnen mit Kindern nur 3 (1,2 %) unter 30 Jahre alt. Im Median waren somit Urologinnen mit Kindern deutlich älter als ihre Kolleginnen ohne Kinder.

Bisher konzentrieren sich die meisten Arbeiten zu dem Thema späte Familiengründung auf die Auswirkungen des fortgeschrittenen Alters der Eltern auf die Fruchtbarkeit, die Schwangerschaft und die Gesundheit des Kindes [9]. Jedoch gibt es auch Arbeiten, die sozialpolitische Aspekte einer späten Familiengründung untersuchen. So fanden Mills et al. in ihrer Analyse heraus, dass zu den wichtigsten Gründen einer späten Geburt des ersten Kindes der Anstieg der Bildung und der Erwerbsbeteiligung von Frauen, der Wertewandel, die Gleichstellung der Geschlechter und das Fehlen einer unterstützenden Familienpolitik zählen [10]. Elternschaft konkurriert nicht nur mit Bildungs- und Beschäftigungswünschen, sondern ist zunehmend zu einer Frage der persönlichen Präferenz geworden. Familiengründung wird häufig in einen Lebensabschnitt aufgeschoben, in dem die Kindererziehung besser mit den persönlichen und beruflichen Zielen der Frauen vereinbar ist [10]. So konnte in einer schwedischen Untersuchung gezeigt werden, dass die Wahrscheinlichkeit ein Kind zu bekommen stieg, wenn die Eltern auf dem Arbeitsmarkt etabliert waren [11]. Auch der Zusammenhang zwischen Bildung der Frauen und Zeitpunkt der Familiengründung ist gut untersucht. Martin et al. konnten zeigen, dass beispielsweise in den USA die zunehmende Bildung der Frauen zu einer Verschiebung des Gebäralters führte [12]. Unsere Daten über Ärztinnen in der Urologie, die ebenfalls gezeigt haben, dass der berufliche Status ein unabhängiger prädiktiver Faktor für eine Mutterschaft ist, stehen hierbei im Einklang mit der Literatur.

Die späte Elternschaft erklärt auch andere in der univariablen Betrachtung signifikant erscheinende Ergebnisse unserer Auswertung. So gaben nur 47 (18,1 %) der 260 Urologinnen mit Kindern an, sich noch in der Weiterbildungszeit zu befinden. Da die Weiterbildungszeit in der Regel direkt nach dem Studium beginnt und zum größten Teil in Kliniken erfolgt, wo auch eine operative Ausbildung stattfindet, sind jüngere Urologinnen, die noch seltener Kinder haben, häufiger in Kliniken und auch operativ tätig. Kinder hatten somit in der multivariablen Analyse keinen Einfluss auf beruflichen Status oder operative Tätigkeit. Und auch auf das Erreichen einer Promotion oder Habilitation hatten Kinder in der multivariablen Analyse keinen negativen Einfluss.

### Hoher Anteil von Teilzeitarbeit

Sehr auffällig erscheint der hohe Anteil von Teilzeitarbeit unter Frauen mit Kindern. Nur 94 (36,2 %) von 260 gaben an, Vollzeit tätig zu sein, während bei Urologinnen ohne Kinder > 90 % (207/224) Vollzeit tätig waren. Dass Kinder ein unabhängiger Einflussfaktor für die Wahrscheinlichkeit von Teilzeitarbeit sind, konnte bereits in einer vorangegangenen Auswertung der Daten gezeigt werden [13]. In unserer Befragung gaben > 75 % der Urologinnen mit Kindern an, dass sie ihre Wochenarbeitsstunden aufgrund von Kindern reduziert haben.

Generell besteht eine gewisse Form der Unvereinbarkeit von Kinderbetreuung und Erwerbstätigkeit [10, 14], die in der soziologischen Literatur ausgiebig adressiert wird. Die Anwesenheit von Kindern am Arbeitsplatz ist in den entwickelten Volkswirtschaften mit wenigen Ausnahmen nicht möglich [15]. Zudem ist eine zeitweise Unterbrechung der Berufstätigkeit rein biologisch unmittelbar vor und nach der Geburt unumgänglich. In unserer Auswertung ist diese Tatsache daran zu erkennen, dass Urologinnen mit Kindern eine signifikante Verlängerung ihrer Weiterbildungszeit von 5,5 Jahre im Median auf 6 Jahre hatten. Auch zeigt der hohe Anteil an Frauen (30 %), die nach der Geburt eines Kindes ihre Arbeitsstätte wechselten, die häufig noch bestehende Unvereinbarkeit von Familie und Beruf. Innerhalb unserer Umfrage hatten die Teilnehmerinnen die Möglichkeit, Kommentare zu hinterlassen.

#### „*Ich habe vielfach Benachteiligung aufgrund meines Geschlechts/meiner Mutterschaft erlebt.“*

Zahlreiche Bemerkungen wie: „Weil ich auf 75 % reduzieren wollte, musste ich meinen Posten als Oberärztin abgeben. Es passte nicht mehr ins Konzept.“ oder „Es ist schade und frustrierend, dass Frauen, die in Teilzeit arbeiten, heutzutage weiterhin in ihrer operativen Tätigkeit nicht unterstützt werden, sondern in den operativen Fächern in die konservativen Bereiche verschoben werden (Konsiliar, Ambulanz …).“, veranschaulichen, dass dieser Wechsel oft nicht nur auf Freiwilligkeit beruht. Unsere Daten legen nahe, dass die bessere Vereinbarkeit von Familie und Beruf häufig in der Praxis gesehen wird, da die Tätigkeit in einer Praxis ein unabhängiger Prädiktor für das Vorhandensein von Kindern war.

### Kinderbetreuung und Erwerbstätigkeit

Jedoch gibt es eine zunehmende Zahl von Publikationen, die darauf hindeuten, dass Erwerbstätigkeit von Frauen und Kindererziehung kombiniert werden können, wenn durch Politik und Institutionen entsprechende Voraussetzungen geschaffen werden [10, 15]. Es ist erwiesen, dass einige sozialpolitische Maßnahmen dem Aufschub des Kinderwunsches effektiv entgegenwirken können. So konnten beispielsweise Rindfuss et al. an einer norwegischen Kohorte zeigen, dass die Verfügbarkeit einer qualitativ hochwertigen Kinderbetreuung einen Einfluss auf den Zeitpunkt der Erstgeburten hat. Denn auch bei Teilzeitarbeit, Gleitzeit und Schichtarbeit bleibt die Herausforderung der Kinderbetreuung während der Berufstätigkeit der Mutter bestehen [15] und untermauerte damit die Ergebnisse von Castles et al. Diese stellten fest, dass das Vorhandensein von Kinderbetreuungseinrichtungen für Kinder unter 3 Jahren ein entscheidender Faktor für den Wiedereintritt von Frauen in das Erwerbsleben ist und somit die Kombination von Elternschaft und Beschäftigung erleichtert [16].

### Situation in anderen deutschsprachigen Ländern

In unserer Studie haben wir uns auf die Situation von Urologinnen in Deutschland konzentriert und Teilnehmerinnen aus anderen deutschsprachigen Ländern von der Analyse ausgeschlossen, um eine homogene Kohorte mit vergleichbaren äußeren Bedingungen zu gewährleisten. Allerdings bleibt die Frage offen, ob sich die Vereinbarkeit von Familie und Beruf im Ausland tendenziell besser darstellt. Diese Fragestellung könnte wertvolle Erkenntnisse für die Verbesserung der Situation in Deutschland liefern.

Zukünftige Befragungen sollten daher die Situation in anderen deutschsprachigen Ländern wie Österreich und der Schweiz genauer untersuchen. Ein Vergleich der unterschiedlichen Modelle und Herangehensweisen könnte zudem helfen, „best practices“ zu identifizieren, die die berufliche und familiäre Belastung von ÄrztInnen effektiv reduzieren.

### Ökonomische Aspekte

Unabhängig von soziologischen bzw. sozialpolitischen Aspekten müssen jedoch auch ökonomische Aspekte der Vereinbarkeit von Familie und Beruf betrachtet werden. Ein Ausscheiden aus dem Erwerbsleben bedeutet einen Verlust an Ausbildungschancen und somit eine Werteminderung des berufsspezifischen Humankapitals [10]. So konnten verschiedene Studien aus Schweden, USA und England zeigen, dass Frauen mit Kindern weniger verdienen [17–19]. In einer neueren US-Studie wies Miller nach, dass ein Jahr verzögerte Mutterschaft das Berufseinkommen von Frauen um 9 %, ihre Berufserfahrung um 6 % und die durchschnittlichen Lohnsätze um 3 % erhöhte [20].

Zur Vereinbarkeit von Familie und Beruf in Gesundheitsberufen in Deutschland gibt es laut einer Metaanalyse von Lukasczik et al. aus dem Jahr 2017 bisher wenig systematische Forschungsergebnisse [21]. In einer Befragung von Krankenhausärztinnen und -ärzten an zwei großen deutschen Universitätskliniken aus dem Jahr 2005 analysierten die Autoren anhand eines strukturierten Fragebogens die Vereinbarkeit von Familie und Beruf anhand eines „work interfering with family conflict scale“ (WIF), welcher bei den Ärztinnen und Ärzten höher als in der deutschen Allgemeinbevölkerung ausfiel [6]. Das Erleben eines Vereinbarkeitskonflikts war mit Burnout, Stress und Kündigungsabsichten korreliert. Ein Unterschied zwischen Ärztinnen und Ärzten konnte jedoch nicht gezeigt werden. Die existierenden Befragungen von Ärzten und Zahnärzten zur Vereinbarkeit von Familie und Beruf in Deutschland geben jedoch ein klares Bild: Im Report Versorgungsforschung gaben 76 % der befragten Ärztinnen und 18 % der Ärzte an, dass sich ihre Weiterbildung durch die Kinderbetreuung verzögert habe [21, 22].

In mehreren Befragungen wurde zudem der Wunsch nach Arbeitszeitreduktion und Optimierung der Kinderbetreuungsmöglichkeiten unter Ärztinnen und Ärzten mit Kindern gezeigt.

Geschlechtergerechtigkeit bedeutet jedoch einen Anspruch auf Gleichstellung aller Geschlechter bei Bildungsniveau, Gesundheit, politischer Handlungsfähigkeit und wirtschaftlicher Teilhabe und Chancen [23]. Dies bedeutet umgekehrt auch eine Gleichstellung bei der Kindererziehung bzw. Care-Arbeit. Schätzungen zufolge könnte unbezahlte Care-Arbeit in Ländern mit hohen Einkommen bis zu 50 % des Bruttoinlandsprodukts (BIP) ausmachen, in Ländern mit niedrigen Einkommen sogar 80 % [24]. Nancy Folbre, Wirtschaftswissenschaftlerin, stellte fest: „Frauen in fast jedem Land verrichten einen großen Anteil der nicht zum Arbeitsmarkt gerechneten Arbeit und insgesamt auch mehr Stunden täglich als Männer“ [24]. Eine gerechte Aufteilung der Care-Arbeit zwischen beiden Elternteilen ist daher essenziell, um eine nachhaltige Vereinbarkeit von Familie und Beruf zu erreichen. Zudem wird durch die fehlende Berücksichtigung der Hausarbeit bei der Berechnung des BIP eine große geschlechterbezogene Datenlücke verursacht. Jakob Hein, ehemaliger Väterbeauftragter der Charité, meinte dazu: „In Deutschland gehen Männer und Frauen als modernes Paar in den Kreißsaal hinein und kommen als 50er-Jahre-Paar wieder heraus“ [25].

Um Gleichberechtigung zu erreichen, müssten Männer ermutigt werden, Elternzeit zu nehmen – und Frauen bestärkt werden, Führungspositionen zu besetzen, sagt die Frauenbeauftragte der Charité, Christine Kurmeyer, in einem Interview zu dem Thema „Karriere in der Medizin“ [26].

Arbeitszeitmodelle, die sich an die Lebensrealität von Eltern anpassen, können dabei unterstützen.

## Fazit für die Praxis

- Unsere Befragung zeigt, dass unter den Urologinnen in Deutschland die Gründung einer Familie häufig mit einer Arbeitszeitreduktion und Beendigung der Klinikkarriere einhergeht.
- Die fortschreitende Feminisierung des Medizinberufs birgt die Gefahr von Versorgungsengpässen, sofern keine Anpassungen im medizinischen Sektor vorgenommen werden.
- In einer Zeit, in der die Geburtenraten sinken und ein Fachkräftemangel herrscht, benötigt unsere Gesellschaft dringend Konzepte, um Eltern die Möglichkeit zu bieten, ohne berufliche Benachteiligung ärztlich tätig zu sein. Die Vereinbarkeit von Familie und Beruf spielt dabei eine entscheidende Rolle, nicht nur für Mütter, sondern auch für Väter. Die Akzeptanz von Teilzeitarbeit trägt dazu bei, dass Eltern als Fachkräfte in Kliniken erhalten bleiben.
- Teilzeitarbeit darf nicht den Karriereaufstieg oder die operative Ausbildung behindern, besonders in chirurgischen Fächern.
- Dies erfordert ein Umdenken in der Gesellschaft als auch unter aktuellen und zukünftigen Führungskräften. Im Wettbewerb der Kliniken um qualifizierte Mitarbeitende werden letztlich auch die Möglichkeit flexibler Arbeitszeitmodelle und Jop-sharing-Konzepte entscheidend sein.

## Data Availability

Die Daten, die die Ergebnisse dieser Studie stützen, sind im Manuskript und dessen ergänzendem Material verfügbar. Detailliertere Daten dieser Studie können auf begründete Anfrage von den korrespondierenden Autoren angefordert werden.
